# Expression of Cytokine Profiles in Human THP-1 Cells during Phase Transition of *Talaromyces marneffei*

**DOI:** 10.3390/pathogens11121465

**Published:** 2022-12-02

**Authors:** Fangyi Shu, Patcharin Thammasit, Kritsada Pruksaphon, Joshua D. Nosanchuk, Sirida Youngchim

**Affiliations:** 1 Department of Microbiology, Faculty of Medicine, Chiang Mai University, Chiang Mai 50200, Thailand; 2 Department of Anatomy, Youjiang Medical University for Nationalities, Baise 533000, China; 3 Department of Medicine, Division of Infectious Diseases, Albert Einstein College of Medicine, New York, NY 10467, USA

**Keywords:** cytokine, macrophage, *Talaromyces marneffei*, phase transition

## Abstract

*Talaromyces marneffei*, a dimorphic fungus, exhibits temperature-dependent growth, existing in a filamentous form at 25 °C and as a yeast at 37 °C. Several studies have highlighted the important roles of macrophages in defense against *T. marneffei* infection. However, the immune responses to the interaction of macrophages with *T. marneffei* cells during phase transition require further investigation. This study reports the expression of cytokine profiles in human THP-1 cells during infection by *T. marneffei***.** THP-1 cells were infected with *T. marneffei* conidia at different multiplicity of infections (MOIs). Surviving conidia transformed into yeasts after phagocytosis by macrophages, and the number of yeasts gradually increased over 36 h. The transcription and secretion levels of pro- and anti-inflammatory cytokines were examined at different times by qRT-PCR and ELISA. Transcription levels of IL-8, IL-12, IL-1β, and TNF-α increased significantly at 12 or 24 h and then slightly decreased at 36 h. In contrast, the transcription levels of IL-6, IL-10, and TGF-β gradually increased at all MOIs. The levels of IL-6 and IL-10 secretion corresponded to their levels of transcription. These results indicated that as the number of intracellular yeasts increased, the infected macrophages first underwent slight M1 polarization before shifting to M2 polarization. This polarization transition was confirmed by the fungicidal ability and the expression of macrophage surface markers. By inducing the M2-type polarization of macrophages, the intracellular *T. marneffei* cells can successfully evade the immune response. Our study provides a novel insight into the immune characterization during the transition of *T. marneffei* infection and could further contribute to possible diagnostic and therapeutic interventions for this infection.

## 1. Introduction

*Talaromyces marneffei*, formerly named *Penicillium marneffei*, is a dimorphic fungus endemic in Northeastern India, Southern China, and Southeast Asian countries [1]. *T. marneffei* infection, also known as talaromycosis, is most probably acquired through environmental inhalation, with the lungs being the most common initial site of infection. Within the endemic regions, the fungus is a major cause of fatal systemic infections in immunocompromised persons [1]. The disease may not manifest in some infected people until they develop immunological dysfunction after exposure to *T. marneffei*. The latency of the disease development suggests that *T. marneffei* can avoid clearance by a normally functioning immune system.

The initial interaction of *T. marneffei* conidia with the phagocytic host cells and the degree of activation of the host’s innate immune responses are critical parameters determining the host’s ability to control the disease [2]. Macrophages are classified as either classically activated cells (M1) or alternatively activated cells (M2) [3]. M1 macrophages are highly anti-microbial and destroy pathogens via NO, ROS production, and degradation in the phagosome. In addition, they secrete pro-inflammatory cytokines, such as IL-12, TNF-α, and IL-6. In contrast, M2 macrophages promote tissue repair and dampen inflammation via the secretion of anti-inflammatory cytokines such as IL-10 and TGF-β [3]. The induction of M2 macrophages is an effective survival strategy for pathogens in the host [4]. Macrophages are essential for mediating the first step of effective antifungal host defense [5]. Macrophages can lose or regain their fungicidal capacity by reversing the polarization phenotype in response to specific microenvironments [6]. For example, macrophages secreted TNF-α, a major pro-inflammatory cytokine involved in early inflammatory events, after being stimulated with *T. marneffei* conidia [7]. In addition, macrophages play an important role in controlling *T. marneffei* growth by killing intracellular yeast cells via secretion of TNF-α [2,8]. In vitro studies and in vivo animal models showed that macrophage-derived cytokines such as TNF-α were important for *T. marneffei* clearance [7,9]. Furthermore, Dong and colleagues found that macrophages can dynamically change inflammatory factors against *T. marneffei* infection, and this phenomenon is necessary to eradicate *T. marneffei* [10]. Importantly, the host immune response against *T. marneffei* infection is mainly mediated by the expression of cytokines to activate the fungicidal activities of macrophages [9]. During talaromycosis, however, phagocytosed conidia of *T. marneffei* can germinate into yeast cells that reside intracellularly within macrophages [11]. These findings suggest that the yeast has the ability to resist phagosomal killing mechanisms [12]. Thus, understanding cytokine profiles during *T. marneffei* phase transition may offer a rationale for monitoring development strategies in talaromycosis.

In the present study, the interaction of macrophages with *T. marneffei* cells was investigated to obtain a first insight into the host response to *T. marneffei* during phase transition. Cytokine profiles in THP-1 monocyte-derived macrophages during the phase transition of *T. marneffei* were determined by ELISA and qRT-PCR. The results of this study contribute to clarifying the development of the initial immune response to *T. marneffei* and provides new insights into immune profiling of the interactions of the fungus with host immune cells. The findings further provide a foundation for the future exploration of the relationship between immunological mechanisms and talaromycosis prognosis.

## 2. Materials and Methods

### 2.1. Preparation of T. marneffei Conidia

*T. marneffei* ATCC 200051 was isolated from a bone marrow sample of a patient with AIDS at the Central Laboratory, Maharaj Nakorn Chiang Mai Hospital, Thailand [13]. *T. marneffei* was maintained by subculture on potato dextrose agar (PDA; Difco, Becton Dickinson, Sparks, MD, USA). *T. marneffei* was then grown on PDA at 25 °C for 6 days. The conidia were collected by adding 5 mL of sterile PBS pH 7.2 containing 0.01% Tween 80 to the culture plate. Conidia were removed by gentle scraping with a sterile cotton swab. The cells were then collected by centrifugation at 8000× *g* for 30 min, and the pellets were washed three times with sterile PBS containing 0.01% Tween 80.

### 2.2. THP-1 Cell Culture and Infection

The human monocyte cell line THP-1 (ATCC TIB-202) was cultured in RPMI 1640 (Gibco; Life Technologies, Carlsbad, CA, USA) supplemented with 10% (*v*/*v*) heat-inactivated FBS (Gibco; Life Technologies, Carlsbad, CA, USA), and penicillin (100 U/mL)/streptomycin (100 µg/mL) under 5% CO_2_ at 37 °C. For differentiation into a macrophage-like cell type, cells (1 × 10^6^ cells/mL) were seeded into a 6-well culture plate with 50 ng/mL phorbol myristate acetate (PMA, Sigma, St. Louis, MO, USA) for 48 h under 5% CO_2_ at 37 °C. After incubation, non-attached cells were removed by aspiration, and the adherent cells were washed with RPMI-1640. The adherent differentiated macrophage-like cells (THP-1 cells) were used for all further experiments. Then, *T. marneffei* conidia suspended in 1.5 mL of RPMI 1640 medium supplemented with 10% (*v*/*v*) heat-inactivated FBS (Gibco; Life Technologies, Carlsbad, CA, USA), penicillin (100 U/mL)/streptomycin (100 µg/mL) were phagocytosed by macrophages for 2 h at MOIs of 1, 5 and 10 at 37 °C. The macrophages that were not infected with conidia served as a control. Next, the macrophages were washed with RPMI 1640 to remove non-internalized conidia. After washing, the macrophages were cultured at different times (1, 8, 12, 24, and 36 h). The infected macrophages were harvested with 0.25% trypsin-EDTA (Gibco; Life Technologies, Carlsbad, CA, USA) for 10 min at 37 °C and then washed three times with PBS. The collected macrophages were subsequently used for flow cytometry, real-time reverse transcription-polymerase chain reaction (qRT-PCR), and killing assays.

### 2.3. Confirmation of the Presence of Yeasts in THP-1 Cells

The monoclonal antibody (MAb) 4D1, which is highly specific against *T. marneffei* cytoplasmic yeast antigens (CYA) [13], was used to confirm the presence of intracellular yeasts in macrophages during infection. The experiment was performed as previously described [13]. Briefly, 1 × 10^6^ THP-1 cells per well were plated on a coverslip-coated 6-well plate and differentiated as described in 2.2. *T. marneffei* conidia were stained with 0.1 mg/mL fluorescein isothiocyanate (FITC, Sigma-Aldrich, St. Louis, MO, USA) in 0.1 M carbonate buffer pH 9.0 overnight at 4 °C with shaking [14]. The labeled conidia were washed three times with PBS containing 0.1% (*v*/*v*) Tween 20 and then used for infection of THP-1 cells in the wells. After 2 h at 37 °C, the non-internalized conidia were removed, and the infected macrophages were cultured for an additional 1, 12, and 36 h at 37 °C. At each time, the cells were fixed with 4% paraformaldehyde for 10 min in cold PBS and then permeabilized with 0.2% Triton X-100 in PBS for 10 min. After 5 washes with PBS, the cells were stained with 0.1 mg/mL of MAb 4D1 for 2 h at 37 °C. The coverslips were washed three times with 2% BSA in PBS and then incubated with the secondary antibody Alexa Fluor 555 conjugated goat anti-mouse IgG (Invitrogen, Eugene, OR, USA) for 1 h at 37 °C. After three washes, the cells were labeled with 0.5 μg/mL 4′,6-diamidino-2-phenylindole (DAPI) for 5 min. The samples were viewed with brightfield and immunofluorescence microscopy using a Nikon DS-Fil (Melville, NY, USA). The experiments were carried out in three independent replicates.

### 2.4. Flow Cytometry for Determining the Percentage of T. marneffei Yeasts in THP-1 Cells

In this study, flow cytometry was applied to determine the percentage of *T. marneffei* yeasts in THP-1 cells infected with conidia at an MOI of 10. The experiments were carried out as described previously [13]. Briefly, the infected THP-1 cells were fixed with 4% paraformaldehyde and lysed with 1% Triton X-100 in PBS. *T. marneffei* cells were washed 5 times with PBS and stained with 0.1 mg/mL MAb 4D1 for 2 h at 37 °C. Then, fungal cells were washed five times and suspended in Alexa Fluor 488 conjugated goat anti-mouse IgG (Invitrogen, Eugene, OR, USA) solution (1:500 dilution) and then incubated for 2 h at 37 °C. After incubation, fungal cells were washed 5 times with PBS and then counted using a flow cytometer (Beckman Coulter, Suzhou, China). The percentages of MAb 4D1-positive cells were determined. The conidia and yeasts incubated with MAb 4D1 acted as negative and positive controls, respectively. The experiments were performed in triplicate.

### 2.5. Real-Time Reverse Transcription-Polymerase Chain Reaction (qRT-PCR)

Total RNA was extracted from the THP-1 cells infected by *T. marneffei* using the Total RNA Isolation NucleoSpin^®^ RNA II (Macherey-Nagel, Duren, Germany) according to the instructions provided by the manufacturer. RNA concentrations were measured by a spectrophotometer (Nanodrop 2000: Thermo Scientific, Waltham, MA, USA). One µg of total RNA was converted into complementary DNA (cDNA) using RevertAid First Strand cDNA Synthesis (Thermo Scientific, Waltham, MA, USA) following the enzyme supplier’s instructions. Real-time RT-PCR assays were carried out using Maxima SYBR Green qPCR Master Mix (Thermo Scientific, Waltham, MA, USA) and the primer sets listed in Table 1. Relative transcription levels were calculated with the ΔΔC_T_ (2^−ΔΔCt^) method [15] using reference gene β-actin for normalization. All measurements were performed in three independent replicates.

### 2.6. Cytokine Determination and Macrophage Killing Assay

To assess cytokine secretion, THP-1 cells were infected with *T. marneffei* conidia at MOI of 10 and incubated for different times (1, 8, 12, 24, and 36 h) at 37 °C. After incubation, the supernatants were collected and kept at −80 °C until the cytokine assays were performed. IL-6 and IL-10 were measured by commercial ELISA kits (Pepro Tech, Cranbury, NJ, USA) following the manufacturer’s instructions. Briefly, 96-well plates were coated with 100 μL of capture antibody overnight at room temperature, and reagent diluent was added for blocking. Then 100 μL of standard or samples were added to wells in triplicate for 2 h at room temperature, and a detection antibody was added to the wells. After washing, the avidin-HRP conjugate was added and incubated for 30 min, and 100 μL of 2,2′-azino-*bis* (3-ethylbenzthiazoline-6-sulfonic acid) (ABTS) was included. After an additional 30 min at room temperature, the absorbance was measured on an ELISA reader (Shimadzu model UV-2401PC, Kyoto, Japan) at 405 nm with wavelength correction set at 650 nm. The assay was repeated three times for at least two independent assays, and the results were expressed as mean OD for each determination.

For the killing assay, the infected macrophages were lysed in 1% Triton X-100. Next, the fungal cell suspension was serially diluted and plated on PDA. After incubation for 48 h at 25 °C, the colony-forming units (CFUs) of *T. marneffei* were counted. The experiments were carried out in three independent replicates.

### 2.7. Immunofluorescence Staining to Identify Macrophage Surface Markers

CD86 (co-stimulatory ligand) and CD206 (mannose receptor, MR) are surface markers of M1 and M2 macrophages, respectively [24,25]. To study the polarization of THP-1 cells during infection, the expressions of CD86 and CD206 were analyzed by immunofluorescence staining. THP-1 cells were infected with an MOI of 10, and infected macrophages were harvested 12, 24, and 36 h after infection at 37 °C. Then, FcRs (Fc receptors) of macrophages were blocked by 1% FBS in PBS on ice for 20 min. After washing once using PBS at 4 °C, the infected macrophages were stained with 10 µg/mL mouse anti-human CD86 or CD206 antibodies (Invitrogen, Rockford, IL, USA) for 45 min at 4 °C. The purified monoclonal antibody 18B7 (anti-cryptococcal glucuronoxylomannan, mouse IgG1) was used as an isotype control [26]. The cells were washed once with PBS containing 1% FBS to eliminate free primary antibodies. The cells were then incubated for 45 min in the dark at 4 °C with 10 µg/mL Alexa Fluor 488-conjugated goat anti-mouse IgG (H + L) (Invitrogen, Eugene, OR, USA). After washing, the cells were fixed with 500 µL of 2% paraformaldehyde in PBS for 15 min at room temperature. The cells were suspended in 500 µL of PBS containing 1% FBS and analyzed using a flow cytometer (Beckman Coulter, Suzhou, China). The experiments were performed in three biological replicates.

### 2.8. Statistical Analysis

The data were analyzed with an unpaired t-test with a significant value of *p* < 0.05. All statistical analysis was performed using SPSS v. 17.0 and GraphPad Prism 8.4.0.

## 3. Results

### 3.1. T. marneffei Shows an Intracellular Transition to Yeast Cells after Phagocytosis

**Figure 1:**
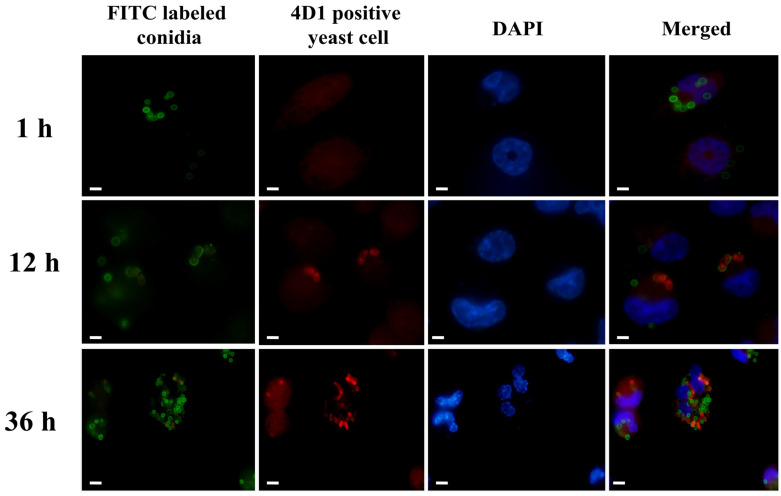
*T. marneffei* yeast in THP-1 cells at different times. THP-1 cells were treated with FITC-labeled *T. marneffei* conidia for 2 h, and then non-phagocytosed conidia were removed. The THP-1 cells were cultured for an additional 1, 12, and 36 h. *T. marneffei* yeast cells were labeled at each time point with MAb 4D1 and Alexa Fluor 555 conjugated goat anti-mouse IgG antibody. From left to right: the conidia stained green with FITC, MAb 4D1-positive yeast cells, the THP-1 nuclei stained blue with DAPI, and a merged channel showing the overlapping of triple images. Bars, 5 μm.

In our study of the intracellular phase transition of conidia to yeast inside THP-1 cells, we showed that *T. marneffei* FITC-labeled conidia were quickly internalized by THP-1 cells by 1 h of interaction. These conidia were bright green, indicative of the mold form. The conidia began to transition into the yeast phase by 12 h, as shown by the red signal of the yeast phase-specific antigen, while the green color labeled the conidial wall. At 36 h, more cells displayed the red signal than green, confirming that the conidia were transformed into yeast cells (Figure 1).

### 3.2. The Percentage of T. marneffei Yeast Cells Increased

**Figure 2:**
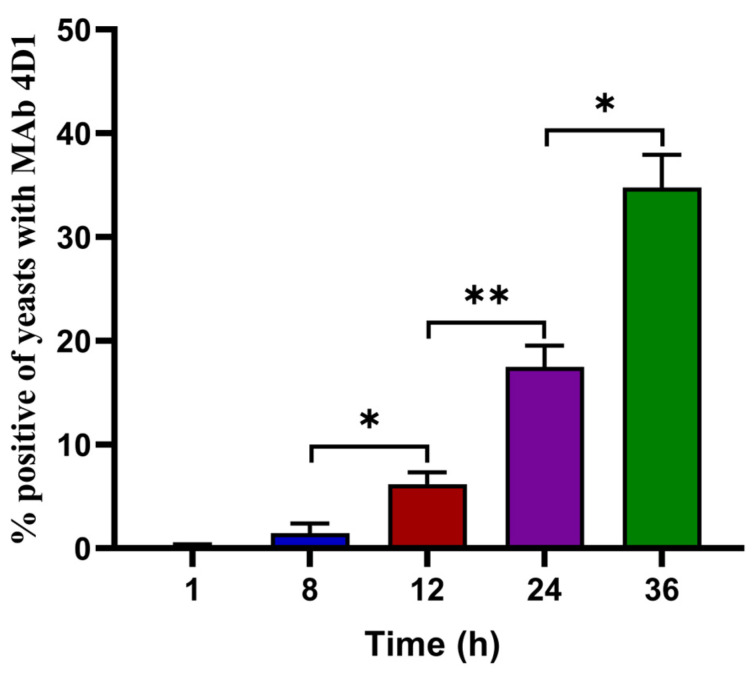
The average percentages of *T. marneffei* yeast cells in THP-1 cells at different times as studied by flow cytometry. After being infected by conidia at an MOI of 10, THP-1 cells were cultured at 37 °C with 5% CO_2_. The average percentages of positive yeast cells  ±  SEM of three sets of independent experiments were shown for each time. Asterisks indicate data that significantly differed (* *p* < 0.05, ** *p* < 0.01) based on Student’s *t*-test (unpaired).

THP-1 cells infected by *T. marneffei* conidia at an MOI of 10 were used for flow cytometry. The percentages of intracellular *T. marneffei* yeasts were determined at 1, 8, 12, 24, and 36 h of co-culture. *T. marneffei* yeast cells were reacted with MAb 4D1 and then captured with fluorochrome antibody conjugate as described in the methods. One of three sets of histograms showed the percentages of MAb 4D1-positive yeast cells at different times (Supplementary Figure S1). The percentages of MAb 4D1 surface-labeled fluorescent yeast cells were initially found after 8 h (1.45%) and then increased significantly to 6.18% by 12 h. At 24 and 36 h of incubation, the percentages of fluorescent-positive yeast cells increased significantly to 17.48% and 37.48%, respectively (Figure 2).

### 3.3. Expression of Cytokines in THP-1 Cells

#### 3.3.1. Expression at Transcription Level

We investigated five pro-inflammatory cytokines (IL-8, IL-12, IL-1β, TNF-α, and IL-6) and two anti-inflammatory cytokines (IL-10 and TGF-β) as produced by infected THP-1 cells using qRT-PCR. Inducible nitric oxide synthase (iNOS) is one of the functional markers of M1 macrophages [22], and the expression level of iNOS was investigated as well.

**Figure 3:**
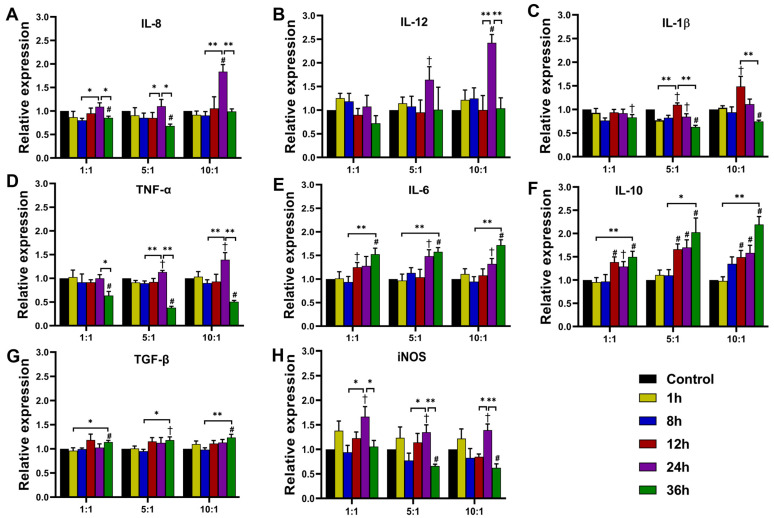
Relative expression of cytokines and iNOS at 1, 8, 12, 24, and 36 h of co-culture with THP-1 cells infected by *T. marneffei* at MOIs of 1, 5, and 10. The transcription levels were analyzed by qRT-PCR and normalized with actin transcription; IL-8 (**A**), IL-12 (**B**), IL-1β (**C**), TNF-α (**D**), IL-6 (**E**), IL-10 (**F**), TGF-β (**G**), and iNOS (**H**). All values are mean ± SEM. Student’s *t*-test (unpaired); * *p* < 0.05, ** *p* < 0.01, ^†^ *p* < 0.05 vs. control, ^#^ *p* < 0.01 vs. control.

At 24 h of co-culture, the expression of IL-8 increased significantly at an MOI of 10 but could not be detected after infection with MOIs of 1 or 5 (Figure 3A). The expression of IL-12 and TNF-α increased significantly at MOIs of 5 and 10 but not at an MOI of 1 (Figure 3B,D). At 12 h, the expression of IL-1β significantly increased at MOIs of 5 and 10 but not at an MOI of 1 (Figure 3C). These results suggested that the expression of cytokines was affected by the number of fungal cells. For example, IL-8, IL-12, IL-1β, and TNF-α expression increased and then decreased (Figure 3A–D). Moreover, iNOS expression was observed to have a similar trend (Figure 3H). At all MOIs, the expression of IL-6, IL-10, and TGF-β gradually increased (Figure 3E–G).

#### 3.3.2. Cytokine Secretion of THP-1 Cells after *T. marneffei* Infection

**Figure 4:**
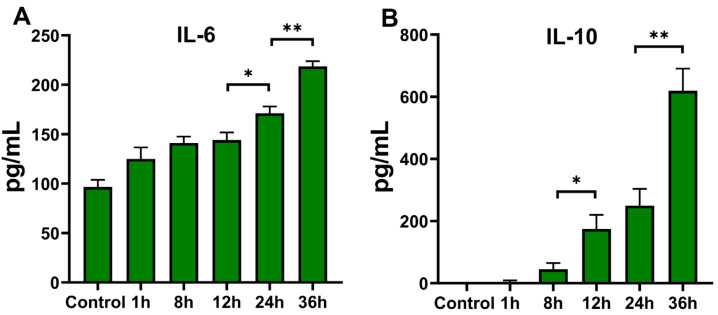
Concentration of cytokines released from THP-1 cells infected by *T. marneffei* conidia at an MOI of 10. The cytokines were measured by ELISA; IL-6 (**A**), IL-10 (**B**). The data are represented as mean ± SEM and analyzed using Student’s *t*-test (unpaired); * *p* < 0.05, and ** *p* < 0.01.

IL-6 is a pro-inflammatory cytokine, and its secretion is typical for an M1-polarized macrophage. Our results reveal that the dynamic transcription level variations of IL-6 were notably distinct from other pro-inflammatory cytokines (as shown in Figure 3). IL-10 is an important marker of M2 macrophage. Therefore, the qRT-PCR results of IL-6 and IL-10 were validated by ELISA using THP-1 cells infected by *T. marneffei* at an MOI of 10. From the 12 to 36 h co-cultures, the concentration of IL-6 gradually increased (Figure 4A). The concentration of IL-10 increased gradually in the 1 to 36 h co-cultures (Figure 4B). Additionally, ELISA results demonstrated that the secretion level of IL-6 and IL-10 was concordant with the transcription level at the MOI of 10 (Figure 3E,F).

Dynamic change in the expression of cytokines in THP-1 cells indicated that as the percentage of intracellular yeast increased, macrophages underwent first a slight M1 polarization before shifting to M2 polarization.

### 3.4. Fungicidal Capacity of THP-1 Cells Changed Dynamically

**Figure 5:**
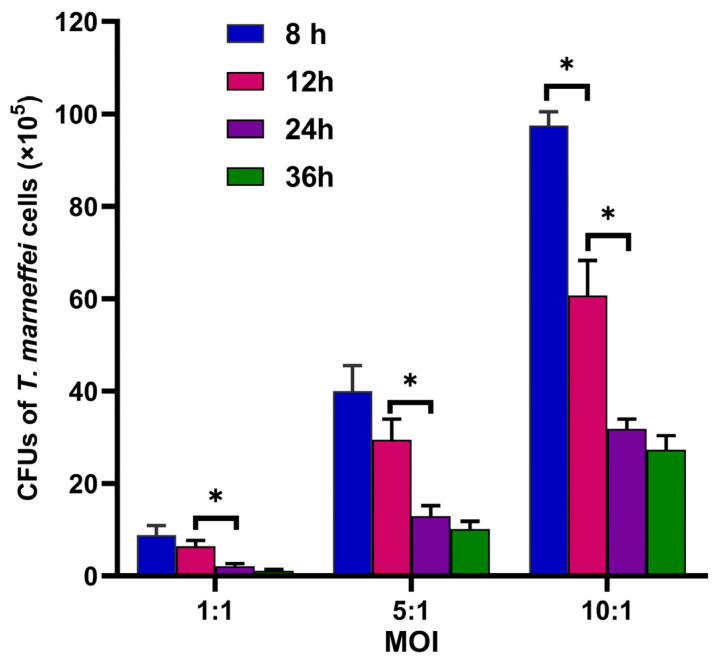
THP-1 cells killing assay. Colony count of *T. marneffei* cells at 8, 12, 24, and 36 h co-culture with THP-1 cells. The experiment was carried out in triplicate, and the data are expressed as mean ± SEM, and Student’s *t*-test was applied for statistical analyses. (* *p* < 0.05).

To confirm the polarity transition of infected macrophages, we investigated their fungicidal capacity at different times of co-culture. As shown in Figure 5, the CFUs of *T. marneffei* cells killed by macrophages decreased steadily over time at all MOIs, and the majority of fungal cells were killed by 36 h, suggesting that macrophages generated potent fungicidal activity. There was a significant decrease in CFUs between the 12 and 24 h co-cultures for all MOIs (1, 5, and 10), but the decreases in *T. marneffei* CFUs at 24 and 36 h were not significant. Based on the significant reduction in CFUs at all MOIs for the 12 to 24 h time points, the macrophages appeared to be highly fungicidal. This finding indicated that a significant number of M1 macrophages were present at 12 to 24 h. Given that there was no significant decrease in CFUs from 24 to 36 h, there were either replication events in the yeast while some fungal cells were being killed or the fungicidal ability of macrophages was attenuated because of a polarity transition from M1 to M2.

### 3.5. Analyzed Expression of the Macrophage Surface Markers CD86 and CD206

**Figure 6:**
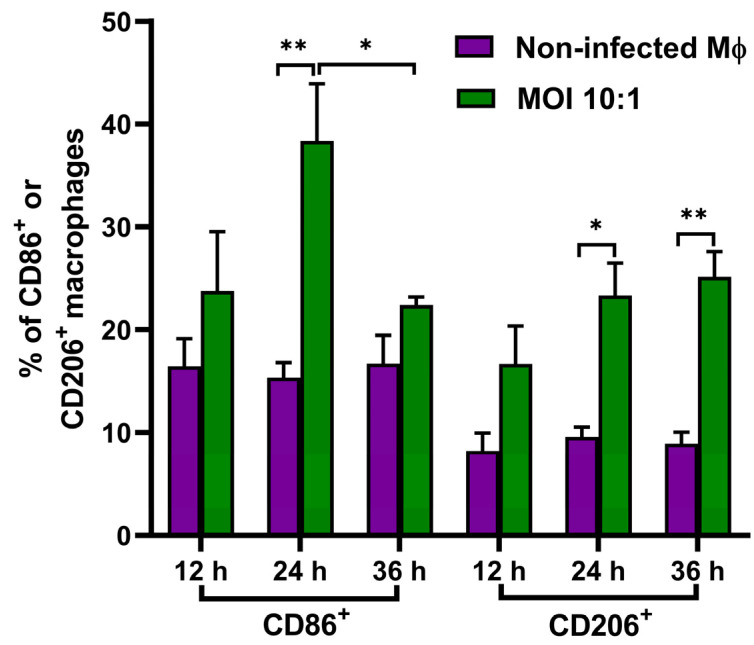
Percentages of M1 or M2 THP-1 cells infected by *T. marneffei* conidia at an MOI of 10. The infected macrophages were stained with 10 µg/mL of mouse anti-human CD86 or CD206 primary antibodies and 10 µg/mL of Alexa Fluor 488 conjugated goat anti-mouse IgG (H + L) secondary antibodies. The stained macrophages were determined by a flow cytometer. All values are mean ± SEM. Student’s *t*-test (unpaired; * *p* < 0.05, ** *p* < 0.01).

To further confirm the polarization status of macrophage cells during infection, we measured macrophage CD86 and CD206 by flow cytometry. At 24 h of THP-1 and *T. marneffei* co-culture, the percentage of CD86^+^ macrophages in infected cells was significantly higher than in uninfected cells (Figure 6). This result indicated that infected macrophages underwent differentiation into the M1 type, endowing them with potent fungicidal capacity. From 24 to 36 h, the percentage of CD86^+^ macrophages declined significantly, suggesting that the polarization of M1 macrophages was reduced. Over this same interval, the percentages of CD206^+^ macrophages in infected cells increased significantly compared with uninfected cells. Hence, the number of M2 macrophages increased as the infection progressed. A representative histogram of three sets shows the percentages of CD86^+^ or CD206^+^ macrophages at different times (Supplementary Figure S2).

Taken together, THP-1 cell polarization status was altered dynamically during infection by *T. marneffei*. The changes correspond with the dynamic alterations in cytokine expression levels (Figure 3 and Figure 4) and macrophage fungicidal ability (Figure 5).

## 4. Discussion

Macrophages play a vital role in protection against *T. marneffei* infection [27]. After phagocytosis of *T. marneffei* conidia, macrophages employ a plethora of killing mechanisms to eliminate the fungal cells. The antifungal processes inside the phagosome include the production of NO and reactive oxygen species, acidification, degradation by hydrolases, and nutrient deprivation [12].

To survive the killing processes, undergo a morphogenic shift from conidia to yeast, and grow inside the macrophage, *T. marneffei* cells adapt to such harsh conditions by a series of strategies such as secreting antioxidant proteins, including superoxide dismutase and catalase-peroxidase and anti-nutrient starvation proteins by induction of the glyoxylate shunt pathway and gluconeogenesis [12]. However, the most important mechanism by which the *T. marneffei* cells establish their survival and initiate replication is the conversion of conidia to the yeast phase. Cytokines play an important role in immune regulation during a *T. marneffei* infection. Herein, we detected the expression of both pro- and anti-inflammatory cytokines in THP-1 cells during the phase transition of *T. marneffei*.

The expression levels of 5 pro-inflammatory cytokines (IL-8, IL-12, IL-1β, TNF-α, and IL-6) were determined. We found that an increase in the percentage of yeast cells resulted in a gradual increase in the expression level of IL-6, while the expression levels of other cytokines first increased and then decreased at MOIs of 5 and 10. The results indicated that the intracellular *T. marneffei* yeasts promoted macrophage polarization toward a classically activated phenotype (M1). M1 macrophage-related pro-inflammatory cytokines may play a critical role in immunological regulation against *T. marneffei* infection. Neutrophils are the first-line cells for defense against fungal infections [27]. IL-8, also known as CXCL8, is involved in recruiting neutrophils to sites of inflammation [28]. IL-12, together with IFN-γ, induces the differentiation of CD4^+^ T cells into the Th1 phenotype [29]. The cytokines produced by Th1 cells, such as IFN-γ, in turn, enhance the fungicidal function of M1 macrophages. IL-1β is important for neutrophil recruitment and superoxide production, and IL-1β was required for the induction of protective Th1 responses during certain disseminated fungal infections [30]. TNF-α is a major regulator in the immune response to fungal infection as it can induce protective Th1 responses against fungal pathogens [31]. IL-6 exerts its pleiotropic effects by modulating the transition from the innate to the adaptive immune response. IL-6 plays a dual role in the differentiation of CD4^+^ T cells. On the one hand, IL-6 induces the differentiation of Th2 cells that secrete IL-4, IL-5, and IL-13, which could promote the development of B cells into plasma cells [32]. On the other hand, IL-6 inhibits the differentiation of Th1 cells [33]. Altogether, the pro-inflammatory cytokines secreted by M1 macrophages generally enhance the antifungal ability of other innate immune cells, such as neutrophils and Th1 cells, to eliminate fungi in the macrophages, while IL-6 may induce a humoral immune response to *T. marneffei* infection.

We also determined the expression levels of two anti-inflammatory cytokines, IL-10 and TGF-β. We found that with the increase in yeast to conidia percentage, the expression levels of IL-10 and TGF-β gradually increased. This result indicated that as the proportion of yeasts in macrophages increased, the polarity of macrophages transitioned from M1 to M2 type by 36 h of co-culture, which was confirmed by the attenuated fungicidal capacity of THP-1 cells (Figure 5), and the expression of CD86 and CD206 of macrophages (Figure 6). Both IL-10 and TGF-β play an important role in dampening immune responses. For instance, TGF-β inhibits T-cell activation and suppresses cytotoxic T lymphocytes (CTL) and Th1 and Th2 lymphocyte differentiation [34]. IL-10 negatively regulates the innate and protective Th1 antifungal activity [31], and macrophage-derived IL-10 can inhibit the differentiation of M1 macrophages [35]. By limiting the excessive inflammatory response, IL-10 can protect tissue from damage. However, the phenotype transition of macrophages from M1 to M2 is helpful in protecting intracellular *T. marneffei* from being killed by other immune cells.

**Figure 7:**
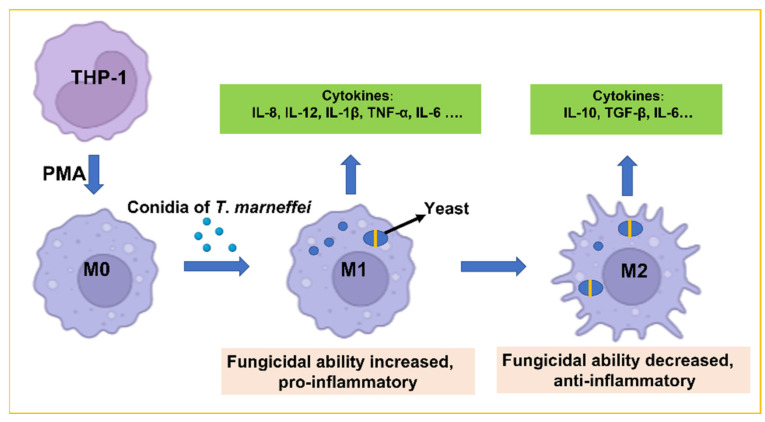
Schematic representation of cytokine profiles in THP-1 cells during the phase transition of *T. marneffei*. After phagocytosis of enough conidia, the macrophages were activated as type M1 and secreted pro-inflammatory cytokines. The surviving *T. marneffei* underwent a phase transition from conidia to yeas. At the same time, M1 macrophages converted to the M2 type. Therefore, the expression of pro-inflammatory cytokines decreased, but the expression of anti-inflammatory cytokines increased.

Interestingly, the transcript levels of both IL-10 and pro-inflammatory cytokines were significantly increased at the 12 h and the 24 h time points. This result indicated that when the macrophages enhanced their fungicidal ability by increasing the expression of pro-inflammatory cytokines, *T. marneffei* activated the expression of IL-10 to promote their survival in the macrophages. The expression of cytokine profiles in THP-1 cells during the phase transition of *T. marneffei* is shown in Figure 7**.**

The human immunodeficiency virus (HIV) mainly invades CD4^+^ T lymphocytes, which leads to severe impairment of the immune function of the organism [36]. Therefore, the role that macrophages play in the fight against *T. marneffei* infection is particularly important for individuals with HIV/AIDS. Understanding the mechanism by which the phenotype transition of macrophages was triggered, or the expression levels of cytokines were changed during the phase transition of *T. marneffei* may provide insights into why immunocompromised individuals, such as in the setting of HIV/AIDS, have such a high risk for disseminated disease. For example, perhaps stopping the phenotype transition of macrophages from M1 to M2 or promoting the expression of pro-inflammatory cytokines, or inhibiting the expression of anti-inflammatory cytokines in macrophages would enhance the anti-*T. marneffei* immunity of HIV/AIDS patients with talaromycosis.

Both innate myeloid cells and adaptive immune cells are involved in the defense against respiratory fungal infection [37]. The innate immune cells involve macrophages, dendritic cells (DC), recruited neutrophils, natural killer cells, and adaptive immune cells consisting of Th1 cells, Th17 cells, and so on. In addition, the innate and adaptive immune cells interact with each other by releasing a series of cytokines and cooperate to effect protective immune responses against respiratory fungal infections. Therefore, there is a limitation to this study. We detected the cytokine profiles produced only by THP-1 cells in vitro, which do not fully represent the complex interplay of immune cells and non-immune cells in vivo. Nevertheless, the present study demonstrated the dual roles of macrophages in protection against *T. marneffei* infection. On the one hand, macrophages kill *T. marneffei* in large numbers and secrete a series of cytokines to regulate immunity against *T. marneffei* infection early after infection. On the other hand, M2 macrophages can protect *T. marneffei* from being killed by other immune cells, such as neutrophils, as the infection progresses.

## 5. Conclusions

Taken together, our findings demonstrated the dynamic changes of macrophage phenotype during the phase transition of *T. marneffei*. THP-1 cells were activated by *T. marneffei* conidia at an early phase. Once dimorphism was established and there were increased numbers of intracellular *T. marneffei* yeasts, macrophages first underwent mild M1 and then M2 polarization. This is an effective strategy for *T. marneffei* to evade the immune response. Our study is the first report that provides a new insight into the macrophage response during the phase transition of *T. marneffei*.

## Figures and Tables

**Table 1:** Primer pairs used in the qRT-PCR analyses.

No	Cytokines	Forward Primer (5′-3′)	Reverse Primer (5′-3′)	Ref.
1	IL-1β	AGCTGGAGAGTGTAGATCCCAA	GGGAACTGGGCAGACTCAAA	[16]
2	IL-6	TGCAATAACCACCCCTGACC	GTGCCCATGCTACATTTGCC	[17]
3	TNF-α	CCCAGGGACCTCTCTCTAATC	ATGGGCTACAGGCTTGTCACT	[18]
4	TGF-β1	GCCCTGGACACCAACTATTGCT	AGGCTCCAAATGTAGGGGCAGG	[19]
5	IL-8	AAGGAACCATCTCACTGTGTGTAAAC	ATCAGGAAGGCTGCCAAGAG	[20]
6	IL-10	GTGATGCCCCAAGCTGAGA	CACGGCCTTGCTCTTGTTTT	[21]
7	IL-12	GCGGAGCTGCTACACTCTCT	GGTGGGTCAGGTTTGATGAT	[22]
8	iNOS	TCCAAGGTATCCTGGAGCGA	CAGGGACGGGAACTCCTCTA	[22]
9	β-actin	ATTGCCGACAGGATGCAGAA	GCTGATCCACATCTGCTGGAA	[23]

## Supplementary Materials

The following supporting information can be downloaded at: https://www.mdpi.com/article/10.3390/pathogens11121465/s1, Figure S1: Percentage of yeast in macrophage; Figure S2: CD86^+^, CD206^+^ macrophage.
