# Supplementary figures and images for: Expression of Cytokine Profiles in Human THP-1 Cells during Phase Transition of Talaromyces marneffei
Source: Pathogens. 2022 Dec 2;11(12):1465. doi: 10.3390/pathogens11121465 (PMC9783046; doi:10.3390/pathogens11121465)

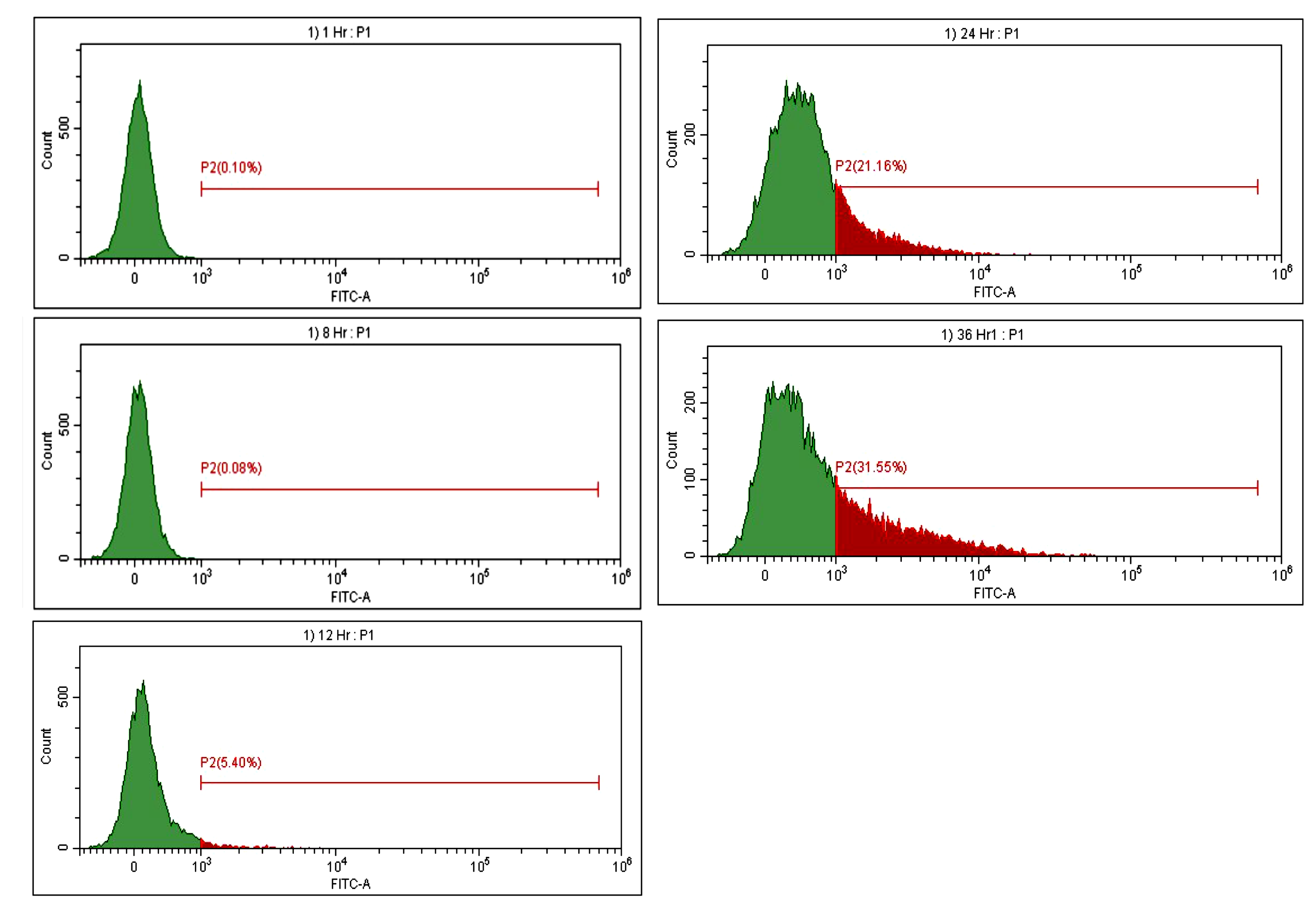

Supplement: Supplementary file 1 [file pathogens-11-01465-s001.zip › pathogens-1996833-supplementary/Figure S1 percentage of yeast in macrophage.tif]

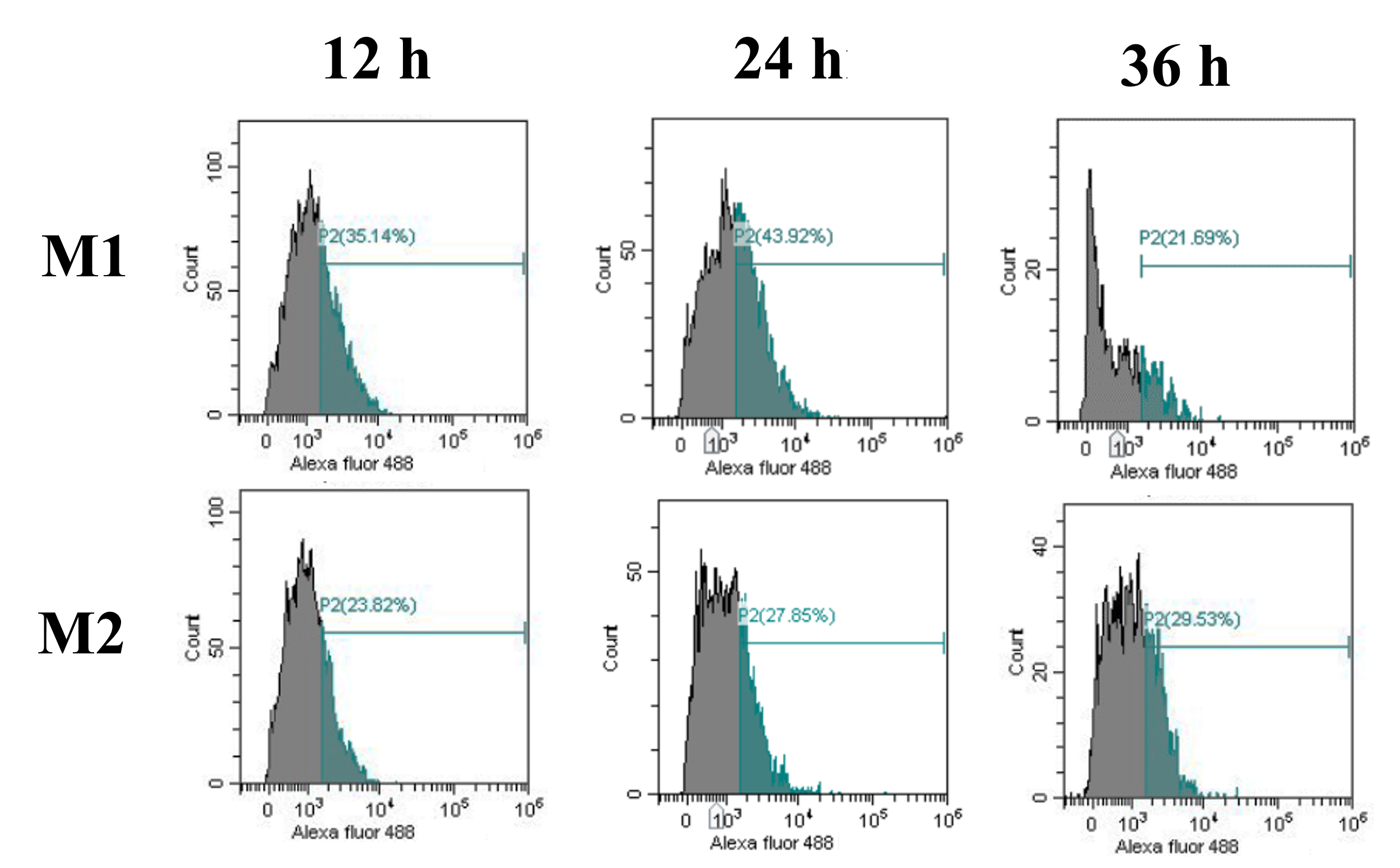

Supplement: Supplementary file 1 [file pathogens-11-01465-s001.zip › pathogens-1996833-supplementary/fIGURE S2 _CD86+, CD206+ macrophage.tif]
